# Visit-to-visit Variability of Blood Pressure and Risk of Stroke: Results of the Kailuan Cohort Study

**DOI:** 10.1038/s41598-017-00380-9

**Published:** 2017-03-21

**Authors:** Haijiang Dai, Yao Lu, Lu Song, Xiaohong Tang, Ying Li, Ruifang Chen, Aijing Luo, Hong Yuan, Shouling Wu

**Affiliations:** 1 Center of Clinical Pharmacology, the Third Xiangya Hospital, Central South University, Changsha, China; 2 Department of Cardiology, the Third Xiangya Hospital, Central South University, Changsha, China; 3 Graduate School, North China University of Science and Technology, Tangshan, China; 4 Department of Cardiology, Kailuan Hospital, North China University of Science and Technology, Tangshan, China

## Abstract

Uncertainty persists regarding the need to address blood pressure (BP) variability in the general population to reduce the heavy burden of stroke. In this cohort study, we prospectively recruited 57,927 participants from southeast of Beijing, who have completed all 3 health examinations between 2006 and 2010. BP variability was defined as the coefficient of variation (CV) across these 3 visits. Over a median follow-up of 3.0 years, we identified 582 first stroke cases. Of these, 489 (84.0%) were ischemic strokes and 94 (16.2%) were hemorrhagic strokes. After multivariable adjustment, the hazard ratios (HR) (95% Confidence Intervals, CI) of comparing participants in the highest versus lowest quartile of CV of systolic blood pressure (SBP) was 1.44 (1.11, 1.87) for any stroke, 1.33 (1.00, 1.77) for ischemic stroke, and 2.17 (1.09, 4.35) for hemorrhagic stroke. Similar results were also observed when the CV of SBP was considered as a continuous exposure variable (per SD increase). Moreover, higher variability of diastolic blood pressure (DBP) was also significantly associated with the risk of any stroke and specifically with hemorrhagic stroke, but not with ischemic stroke. In conclusion, higher visit-to-visit BP variability might be an important target to reduce stroke risk, particularly for hemorrhagic stroke.

## Introduction

Stroke is a major healthcare concern and a serious economic burden on society, especially in developing countries^1, 2^. It has been reported that 63% of ischemic strokes and 80% of hemorrhagic strokes take place in developing countries^2^. In China, stroke has become the leading cause of long-term disability and mortality^3^. Hypertension is typically considered to be the most important modifiable risk factor for stroke. An overwhelming amount of evidence now shows that treatment of high BP is effective in reducing stroke morbidity and mortality^4, 5^. However, although trends in hypertension treatment and control rates have significantly improved, the prevalence of stroke remains substantial^6–8^. Hence, more studies are urgently needed to explore additional modifiable risk factors for stroke.

Recently, a study by Muntner *et al.* ^9^ showed that BP variability, especially variability of SBP, significantly predicts the risk of stroke in hypertensive participants, independent of mean BP. Similar results were also found in postmenopausal women by Shimbo *et al.* ^10^. These compelling findings raise the question whether BP variability, in addition to mean BP, should be a target for preventing the occurrence of stroke. Notably, most previous studies focused on patients with chronic diseases; however, the correlation of BP variability and stroke in the general population is obscure. Whether it is necessary to pay attention to BP variability even in the general population to reduce the stroke burden is still uncertain. Moreover, it is also important to confirm whether these associations differ according to stroke subtype, considering the substantial difference in the distribution of stroke subtypes in different countries^2^. In this study, we prospectively performed a cohort study to examine the potential associations between BP variability and the risk of stroke and each of its subtypes in a Chinese population.

## Methods

### Study population

The Kailuan study^11, 12^ is a prospective population-based cohort study conducted in the Kailuan Community in Tangshan, a large modern city located in southeast of Beijing. The Kailuan Community is a fully functional community owned and administrated by the Kailuan Group Corporation. There are 11 hospitals responsible for healthcare of this community. In 2006–2007, the community began its first health examinations for in-service and retired workers. A total of 101 510 participants (81 110 men; age range 18–98 years) agreed to participate and completed the questionnaires and clinical examinations. In this population, hypertension is highly prevalent with a morbidity of 44.0%, and the proportion of the elderly population (≥60 years) is 23.5%. These participants were then followed through 2010, during which time they completed repeated questionnaires and clinical and laboratory examinations every 2 years. As a result, 57 927 participants completed all 3 health examinations and were included in our present study. However, the participants were excluded if their blood pressure information was available for fewer than 3 visits (n = 1888), if they had a history of stroke at baseline (n = 794) or if they experienced a stroke during the time covering the 3 visits (n = 595). Finally, 54 650 eligible participants were included in the current analyses of stroke risk.

The study was approved by the Ethics Committees of the Kailuan General Hospital. All experiments in this study were performed according to the guidelines from the Helsinki Declaration and written informed consent was obtained from all participants.

### Definition of blood pressure variability

BP was measured by certified nurses with an appropriately sized cuff and a corrected mercury sphygmomanometer. Each participant was required to refrain from cigarette smoking, drinking tea or coffee, and exercising for at least 30 min before the BP measurements. During the measurements, the participants sat with their arms and feet flat, and their upper arms at the height of their heart. SBP and DBP were recorded upon auscultation of the first and fifth Korotkoff sounds, respectively. Each participant was measured three times, with a 30 s interval. If two measurements differed by >5 mmHg, BP was re-measured. The final BP was calculated as the mean of the three measurements.

As previously reported^13^, BP variability was defined as the CV (calculated as standard deviation (SD)/mean BP × 100%) of the BP between visits. Other measures of BP variability, such as SD, average real variability (ARV), and variability independent of mean (VIM), were also calculated. The SD, ARV, and VIM of BP were highly correlated with the CV of BP (r = 0.98, r = 0.90 and r = 0.99 for SBP, respectively; r = 0.98, r = 0.88 and r = 1.00 for DBP, respectively) and were therefore not used to assess the ability of BP variability to predict stroke risk (presented only in the supplementary materials).

### Data collection of potential covariates

For included participants, demographic data on age, gender, education level, income level, and lifestyle information including cigarette smoking, alcohol consumption, and physical activity were obtained from questionnaires via face-to-face interviews. The level of education was stratified into “Illiteracy/Primary”, “High school”, and “College or higher”. The level of income (in Chinese Yuan, ¥) was reported as “<1000 ¥”, “1000–3000 ¥”, and “≥3000 ¥”. Physical activity was defined as the frequency of physical activity (for 20+ min) during leisure time and was scored as never, occasionally (1–3 times per week), and frequently (≥4 times per week). Each participant’s history of physician-diagnosed diseases, such as hypertension, diabetes, and myocardial infarction, was also self-reported using a yes/no response format through the questionnaires.

Height and body weight were measured while the subjects were wearing lightweight clothing, and body mass index (BMI) was calculated as body weight divided by height squared. Resting heart rate was measured using a 10-s 12-lead electrocardiography after the participant had rested in the supine position for 5 min. Venous blood sampling was performed after overnight fasting for 8–12 h, and blood glucose, triglycerides (TG), high-density lipoprotein (HDL), low-density lipoprotein (LDL), uric acid and high-sensitivity C-reactive protein (hs-CRP) levels were determined using standard laboratory methods. Dyslipidemia was defined as LDL ≥ 3.4 mmol/L, or HDL < 1.0 mmol/L, or TG ≥ 1.7 mmol/L, or current treatment with lipid-lowering therapy^14^.

### Follow-up and stroke assessment

After the first 3 visits, all participants were then followed up for incident stroke until December 31, 2013 or until the event of a stroke or death. The follow-ups were performed by hospital physicians, research physicians, and research nurses. For the participants without face-to-face follow-ups, their outcome information was obtained by referring to death certificates from provincial vital statistics offices, discharge summaries from the 11 hospitals, or medical records from medical insurance companies^12, 15^.

As the primary outcome, stroke was diagnosed according to World Health Organization (WHO) criteria^16^ combined with brain computed tomography (CT) or magnetic resonance (MR), and classified as either ischemic or hemorrhagic stroke. The criteria were consistent across all 11 hospitals. All stroke records were reviewed and validated by the Data Safety Monitoring Board and Arbitration Committee for Clinical Outcomes.

### Statistical analyses

The characteristics of the participants included in the current study were analyzed after the participants were stratified into quartiles into the following groups according to the CV of SBP: the Q1 group, including participants with CV < 4.65%; the Q2 group, including participants with a CV ranging from 4.65% to <7.45%; the Q3 group, including participants with a CV ranging from 7.45% to <10.62%; and the Q4 group, including participants with a CV ≥ 10.62%. Categorical variables were expressed as percentages (%), and continuous variables were expressed as the means ± SD. Abnormally distributed variables (TG and hs-CRP) were log-transformed to normality before the analysis. Pearson’s χ^2^ test was used to compare differences between groups for categorical variables, and one-way ANOVA was used for continuous variables. The Kaplan–Meier method was used to calculate the cumulative incidence of stroke events in each quartile group of CV of BP. A series of cox regression models were used to calculate multivariable-adjusted HR (95% CI) for stroke risk associated with a quartile of CV of BP, with the lowest quartile serving as the reference. All statistical analyses were conducted using SPSS software (version 22.0). Associations were considered significant at *P* < 0.05.

## Results

The characteristics of the study participants according to quartiles of CV of SBP are presented in Table 1. Older persons were associated with being in a higher quartile of CV of SBP, whereas men were more likely to be in the lower quartile of CV of SBP. Participants with higher CV of SBP were less likely to report current drinking or current smoking, and these individuals had higher hs-CRP, SBP and DBP levels before follow-up and were more likely to have hypertension, diabetes mellitus, a history of myocardial infarction and to use antihypertensive medication. Furthermore, the levels of education, income, BMI, heart rate, and the frequency of physical activity were also significant different among the CV quartiles. However, no significant difference was observed among the CV quartiles for serum uric acid levels, and the prevalence of dyslipidemia and family history of stroke.

**Table 1:** Clinical characteristics of the study population according to the quartiles of coefficient of variation of systolic blood pressure.

	Quartiles of CV of SBP				*P* value
	Q1 (<4.65%)	Q2 (4.65% to <7.45%)	Q3 (7.45% to <10.62%)	Q4 (≥10.62%)	
	*N* = 13665	*N* = 13659	*N* = 13665	*N* = 13661	
Male (%)	10878 (79.6%)	10390 (76.1%)	10464 (76.6%)	10325 (75.6%)	<0.001
Age, years	51.70 ± 11.80	51.43 ± 12.01	52.81 ± 11.99	55.04 ± 11.85	<0.001
BMI, kg/m^2^	25.20 ± 3.31	25.06 ± 3.41	25.14 ± 3.39	25.08 ± 3.40	0.004
Alcohol drinking, n (%)					<0.001
Never	8336 (61.2%)	8772 (64.4%)	8827 (64.7%)	9123 (67.0%)	
Past	103 (0.8%)	86 (0.6%)	70 (0.5%)	86 (0.6%)	
Current	5191 (38.1%)	4772 (35.0%)	4737 (34.7%)	4415 (32.4%)	
Smoking, n (%)					<0.001
Never	8112 (59.5%)	8320 (61.0%)	8425 (61.8%)	8624 (63.3%)	
Past	656 (4.8%)	676 (5.0%)	598 (4.4%)	642 (4.7%)	
Current	4868 (35.7%)	4637 (34.0%)	4612 (33.8%)	4359 (32.0%)	
Education level, n (%)					<0.001
Illiteracy/Primary	899 (6.6%)	892 (6.6%)	986 (7.2%)	1176 (8.6%)	
High school	11064 (81.2%)	10895 (80.0%)	11034 (81.0%)	11127 (81.7%)	
College or higher	1658 (12.2%)	1827 (13.4%)	1601 (11.8%)	1316 (9.7%)	
Income level, n (%)					0.022
<1000 ¥	6242 (46.8%)	6238 (46.7%)	6124 (45.8%)	6039 (45.2%)	
1000–3000 ¥	5380 (40.3%)	5398 (40.4%)	5436 (40.6%)	5616 (42.0%)	
≥3000 ¥	1726 (12.9%)	1730 (12.9%)	1813 (13.6%)	1710 (12.8%)	
Physical activity, n (%)					<0.001
Never	4688 (34.4%)	4606 (33.8%)	4510 (33.1%)	4335 (31.8%)	
Occasionally	6975 (51.2%)	7152 (52.5%)	7133 (52.3%)	7261 (53.3%)	
Frequently	1971 (14.5%)	1873 (13.7%)	1993 (14.6%)	2029 (14.9%)	
Heart rate, bpm	73.33 ± 10.21	73.12 ± 10.27	73.12 ± 10.28	73.58 ± 10.93	0.001
Serum uric acid, mmol/L	294.39 ± 89.31	293.01 ± 87.99	292.49 ± 88.95	292.28 ± 88.41	0.203
Lg hsCRP, mg/L	0.10 ± 0.49	0.10 ± 0.49	0.11 ± 0.48	0.14 ± 0.49	<0.001
SBP, mmHg	129.22 ± 15.70	128.26 ± 18.19	130.36 ± 18.39	135.70 ± 23.31	<0.001
DBP, mmHg	84.03 ± 9.70	83.35 ± 10.51	84.05 ± 10.51	85.79 ± 12.38	<0.001
Hypertension, n (%)	5797 (42.4%)	5469 (40.0%)	6198 (45.4%)	7702 (56.4%)	<0.001
Diabetes mellitus, n (%)	1364 (10.0%)	1352 (9.9%)	1422 (10.4%)	1754 (12.8%)	<0.001
Dyslipidemia, n (%)	5780 (42.6%)	5633 (41.5%)	5714 (42.1%)	5815 (42.9%)	0.108
History of MI, n (%)	161 (1.2%)	129 (0.9%)	182 (1.3%)	195 (1.4%)	0.002
Family history of stroke, n (%)	527 (3.9%)	532 (3.9%)	463 (3.4%)	486 (3.6%)	0.078
Use of antihypertensive, n (%)	1342 (9.8%)	1382 (10.1%)	1460 (10.7%)	2038 (14.9%)	<0.001

BMI: body mass index; Lg hs-CRP: high-sensitivity C-reactive protein after logarithmic transformation; SBP: systolic blood pressure; DBP: diastolic blood pressure; MI: myocardial infarction.

Over a median follow-up of 3.0 years (range: 2 days to 3.9 years), we identified 582 first stroke cases. Of these, 489 (84.0%) were ischemic strokes and 94 (16.2%) were hemorrhagic strokes. Table 2 shows the distribution of stroke events across the CV quartiles. The cumulative incidence of stroke and its subtypes progressively increased at higher levels of CV of SBP. After full multivariable adjustment, participants in the highest versus lowest quartile of CV of SBP had a significantly higher risk for any stroke, ischemic stroke, and hemorrhagic stroke. The HRs (95% CI) were 1.44 (1.11, 1.87), 1.33 (1.00, 1.77), and 2.17 (1.09, 4.35), respectively. Similar results were also observed when CV of SBP was considered as a continuous exposure variable (per each 4.7% increase, Table 2) or when variability of SBP was calculated using SD, ARV, and VIM (Table S1 in the supplementary data). Moreover, the associations between variability of DBP and the risk of stroke and its subtypes were also calculated in the current analysis (Tables S2 and S3 in the supplementary data). Higher variability of DBP was significantly associated with the risk of any stroke and specifically with hemorrhagic stroke, but not with ischemic stroke.

**Table 2:** Hazard ratios for stroke by quartiles of coefficient of variation of systolic blood pressure and per SD increases in the coefficient of variation.

	Quartiles of CV of SBP				CV of SBP (per SD increase)
	Q1 (<4.65%)	Q2 (4.65% to <7.45%)	Q3 (7.45% to <10.62%)	Q4 (≥10.62%)	
Any stroke					
Case (%)	116 (0.86)	127 (0.94)	137 (1.01)	202 (1.48)	
Model 1	1.00 (ref)	1.12 (0.87, 1.45)	1.17 (0.91, 1.51)	1.50 (1.18, 1.89)*	1.22 (1.14, 1.31)**
Model 2	1.00 (ref)	1.20 (0.90, 1.59)	1.20 (0.91, 1.59)	1.51 (1.16, 1.96)*	1.21 (1.12, 1.31)**
Model 3	1.00 (ref)	1.19 (0.90, 1.58)	1.20 (0.91, 1.59)	1.44 (1.11, 1.87)*	1.17 (1.08, 1.26)**
Ischemic stroke					
Case (%)	101 (0.75)	106 (0.79)	118 (0.87)	164 (1.20)	
Model 1	1.00 (ref)	1.08 (0.82, 1.43)	1.17 (0.90, 1.53)	1.38 (1.07, 1.79)*	1.18 (1.09, 1.28)**
Model 2	1.00 (ref)	1.17 (0.86, 1.59)	1.21 (0.90, 1.63)	1.39 (1.05, 1.85)*	1.17 (1.07, 1.27)**
Model 3	1.00 (ref)	1.16 (0.86, 1.58)	1.20 (0.89, 1.62)	1.33 (1.00, 1.77)*	1.13 (1.04, 1.23)*
Hemorrhagic stroke					
Case (%)	15 (0.11)	21 (0.16)	20 (0.15)	38 (0.28)	
Model 1	1.00 (ref)	1.40 (0.70, 2.78)	1.24 (0.62, 2.50)	2.28 (1.22, 4.24)*	1.38 (1.21, 1.57)**
Model 2	1.00 (ref)	1.39 (0.64, 3.03)	1.21 (0.55, 2.66)	2.36 (1.18, 4.72)*	1.40 (1.22, 1.60)**
Model 3	1.00 (ref)	1.40 (0.64, 3.05)	1.27 (0.58, 2.78)	2.17 (1.09, 4.35)*	1.34 (1.14, 1.57)**

Case (%): number of events (cumulative incidence%).

Model 1: adjusted for age, gender, and body mass index.

Model 2: further adjusted for current smoking, current drinking, education level, income level, physical activity, heart rate, diabetes mellitus, dyslipidemia, serum uric acid, high-sensitivity C-reactive protein after logarithmic transformation, history of MI, family history of stroke, and the use of antihypertensive drugs.

Model 3: further adjusted for mean of systolic blood pressure and mean of diastolic blood pressure.

Per SD increase in the CV of SBP = 4.7%.

*P < 0.05, **P < 0.001.

**Figure 1:**
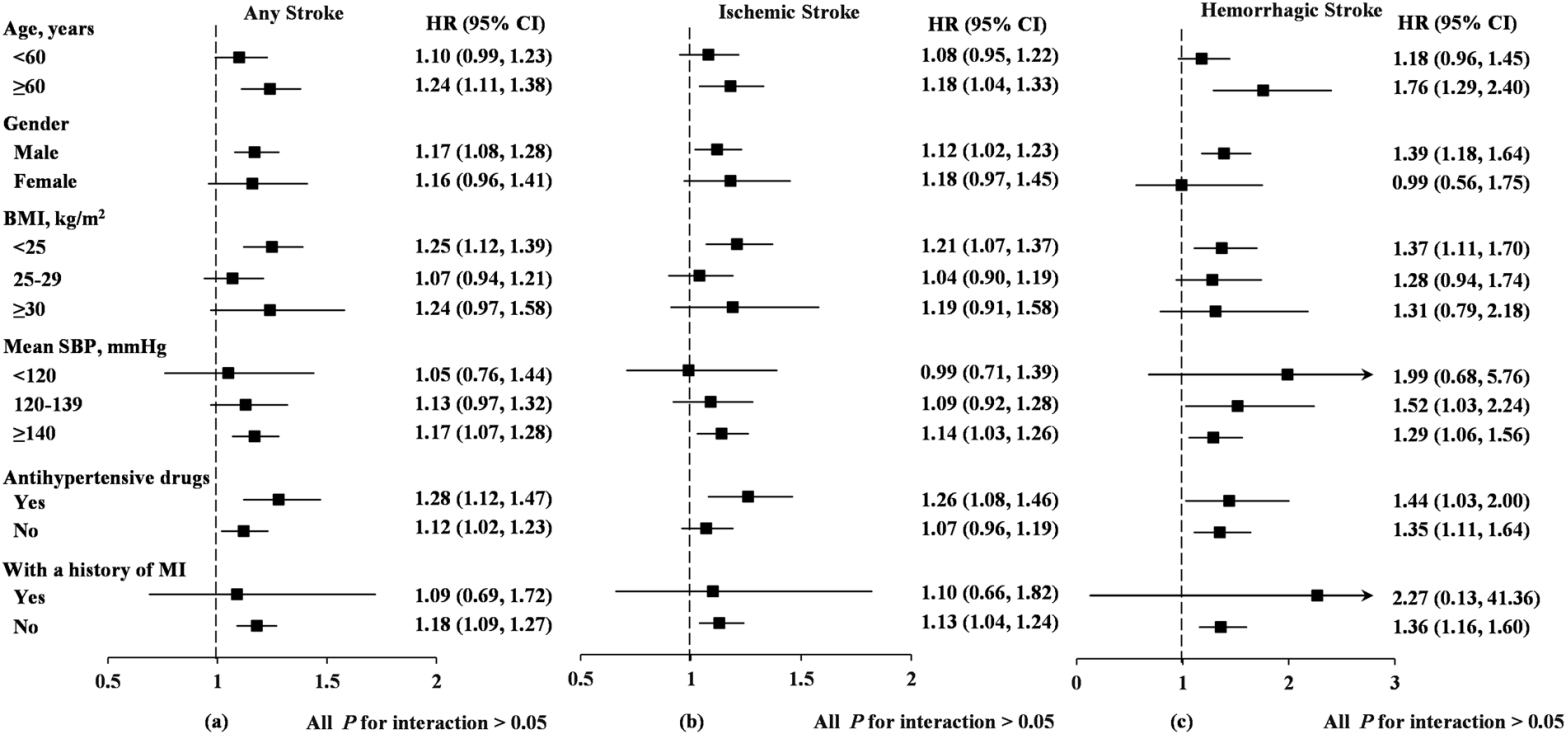
Hazard ratios for stroke outcomes associated with per SD increases in the CV of SBP in selected subgroups. Data were adjusted for age, gender, body mass index, current smoking, current drinking, education level, income level, physical activity, heart rate, diabetes mellitus, dyslipidemia, serum uric acid, high-sensitivity C-reactive protein after logarithmic transformation, history of MI, family history of stroke, and the use of antihypertensive drugs, mean of systolic blood pressure and mean of diastolic blood pressure.

To determine the effect of potential confounding factors, the associations between CV of SBP and stroke events were further investigated among subgroups defined by age, gender, BMI, mean SBP, use of antihypertensive drugs, and history of MI (Fig. 1). As a result, all interaction *P* values were greater than 0.05 in each subgroup (interaction P values = 0.944, 0.778, 0.434, 0.594, 0.150, and 0.445, respectively, for any stroke; 0.497, 0.746, 0.313, 0.396, 0.111, and 0.646, respectively, for ischemic stroke; and 0.171, 0.270, 0.611, 0.400, 0.887, and 0.436, respectively, for hemorrhagic stroke). These data suggest that CV of SBP is an independent risk factor for the incidence of stroke and its subtypes.

## Discussion

In this large prospective cohort study of 54 650 participants, we found close associations between SBP variability and the risk of a stroke event, particularly hemorrhagic stroke. These associations remained after further adjusting for mean BP and were consistent across several subgroups. A significant association was also found between DBP variability and the risk of hemorrhagic but not ischemic stroke. These data add to the growing body of evidence regarding the prognostic value of BP variability as a stroke risk factor, notably for hemorrhagic stroke.

Over the past decade, an accumulating number of reports have documented a relationship between visit-to-visit variability of BP and the risk of stroke. In a secondary analysis of four independent cohorts of patients with TIA and minor stroke, Rothwell *et al.* ^17^ identified a significant association between SBP variability and the risk of stroke. When compared the highest and lowest decile of CV of SBP, the multivariable-adjusted HR (95% CI) for stroke was 3.82 (2.54, 5.73) in the UK-TIA aspirin trial, 3.51 (1.56, 7.93) in the atenolol group and 3.25 (1.32, 8.00) in the amlodipine group in the ASCOT-BPLA trial, 2.22 (1.52, 3.22) in the European Stroke Prevention Study, and 3.41 (1.62, 7.19) in the Dutch TIA trial. Enayet *et al.* ^18^ conducted another secondary analysis of the ANBP2 trial and found that in elderly hypertensive patients, when the participants were divided according to SBP variability, the highest decile group was associated with a 2.78-fold higher risk than the lowest decile. Consistent with these findings, the positive association of SBP variability with stroke was reported in a meta-analysis by Diaz *et al.* ^19^ as well as several recent studies in hypertensive populations^9, 20, 21^. Chang *et al.* ^22^ also found that higher SBP variability was independently associated with stroke in patients with chronic kidney disease. However, most of these studies were conducted in individuals with chronic diseases. Whether examination of SBP variability in the general population is warranted requires further research.

Additionally, these previous findings contrast with the results of a study that explored the association between BP variability and the risk of cardiovascular events and all causes of mortality^23^. That study showed that long-term SBP variability was independently associated with a higher risk of subsequent mortality and myocardial infarction but not stroke^23^. Over a median follow-up of 12 years, Schutte *et al.* ^24^ found that SBP variability did not predict any fatal or nonfatal cardiovascular events, including stroke. In another study, Tully *et al.* ^25^ also showed that there was no association between SBP variability and the incidence of stroke in people aged ≥65 years. Given these controversial results, we conducted an analysis to explore the relationship between SBP variability and the risk of stroke in a large-scale Chinese general population. Our analysis showed that the highest quartile of CV of SBP was associated with a 1.44-fold higher adjusted risk of stroke than the lowest quartile.

The clinical implications of DBP variability are usually considered to be weak for stroke. In a prospective study of 632 consecutive ischemic stroke patients, Lau *et al.* ^13^ found that an increase in DBP variability did not predict recurrent nonfatal strokes. This finding was consistent with those reported in some previous studies. For example, in the PROSPER study, no association was found between visit-to-visit variability in DBP and the risk of stroke^26^. Additionally, in the UK-TIA cohort, DBP variability was not associated with the subsequent incidence of stroke, and an association was found only in the highest decile group in the ASCOT-BPLA trial^17^. In light of these findings, DBP variability does not appear to be a valuable therapeutic target for stroke. However, the effect of DBP variability on stroke was similar to that of SBP variability in our study, particularly for hemorrhagic stroke.

Surveys of stroke epidemiology have shown that the distribution of stroke subtypes is quite different among various regions and countries^2, 27^. For example, the prevalence of hemorrhagic stroke was obviously greater in China than in other countries^2, 28^. Hence, subtype-dependent associations between BP variability and the risk of stroke may indicate different importance of BP variability in different regions and countries. Over the past few years, the associations between BP variability and the risk of stroke subtypes have been reported in several studies^10, 17, 22^. In the post-hoc analysis of the ASCOT-BPLA trial, Rothwell *et al.* ^17^ reported that SBP variability was better at predicting ischemic than hemorrhagic strokes in treated hypertensive participants. In contrast, Chang *et al.* ^22^ found that the highest versus the lowest quintile of the CV of SBP was only associated with higher adjusted rates of hemorrhagic stroke but not ischemic stroke. However, Shimbo *et al.* ^10^ showed that no difference existed between SBP variability and stroke according to subtype (ischemic versus hemorrhagic). In our study, we demonstrated a clearly closer association between BP variability and hemorrhagic stroke than its association with ischemic stroke. Considering the serious burden of hemorrhagic stroke in China, these results indicate that greater focus on BP variability in the general Chinese population may be warranted. However, additional studies are needed to determine whether lowering BP variability is effective in reducing stroke risks.

Our study has several limitations. First, some confounding factors including use of aspirin, medication adherence, length of medication use, and length of diagnoses were not included in our analysis due to data missing. However, the proportion of data missing in included variables were very limited, which would strengthen the credibility of our results. Second, visit-to-visit variability of BP was calculated using three BP values from physical examinations with an interval of 2 years, which may affect the repeatability of BP variability. Therefore, we performed three BP measurements every time instead of just one follow-up measurement^29^ to improve the repeatability of BP variability in our study. Finally, 3 years is relative short for the follow-up of stroke. However, it is should be noted that the number of stroke events is really considerable due to a large number of individuals and a high stroke morbidity in our country^2^. Actually, 582 stroke cases are sufficient enough to draw a reliable conclusion.

In conclusion, our results demonstrate that higher visit-to-visit variability of BP, independent of mean BP, was significantly associated with a higher risk of stroke. The association was particularly significant for hemorrhagic stroke. Higher visit-to-visit variability of BP might be an important target to monitor for decreasing the risk of stroke even in the general population.

## Electronic supplementary material

Supplementary Tables
