# Supplementary material for: Visit-to-visit Variability of Blood Pressure and Risk of Stroke: Results of the Kailuan Cohort Study
Source: Sci Rep. 2017 Mar 21;7:285. doi: 10.1038/s41598-017-00380-9 (PMC5428298; doi:10.1038/s41598-017-00380-9)
Supplement: Supplementary file 1 — Supplementary Tables [file 41598_2017_380_MOESM1_ESM.pdf]

Supplementary Tables

Visit-to-visit Variability of Blood Pressure and Risk of Stroke: Results of the Kailuan Cohort Study

Haijiang Dai[1, 2], Yao Lu[1], Lu Song[3], Xiaohong Tang[2], Ying Li[1], Ruifang Chen[1], Aijing Luo[1], Hong Yuan[1, 2] and Shouling Wu[4]

[1] Center of Clinical Pharmacology, the Third Xiangya Hospital, Central South University, Changsha, China.

[2] Department of Cardiology, the Third Xiangya Hospital, Central South University, Changsha, China.

[3] Graduate School, North China University of Science and Technology, Tangshan, China.

[4] Department of Cardiology, Kailuan Hospital, North China University of Science and Technology, Tangshan, China.

TABLE S1. Hazard ratios for stroke with per SD increases in other indicators of variability of systolic blood pressure

	**Any stroke**	**Ischemic stroke**	**Hemorrhagic stroke**
**SD (+6.726 mmHg)**			
**Model 1**	1.25(1.19, 1.31)**	1.23(1.16, 1.30)**	1.32(1.22, 1.43)**
**Model 2**	1.23(1.17, 1.30)**	1.20(1.13, 1.29)**	1.32(1.21, 1.44)**
**Model 3**	1.16(1.08, 1.24)**	1.13(1.04, 1.22)*	1.26(1.11, 1.44)**
**ARV (+9.223 mmHg)**			
**Model 1**	1.18(1.13, 1.23)**	1.16(1.09, 1.23)**	1.24(1.15, 1.32)**
**Model 2**	1.16(1.10, 1.23)**	1.13(1.05, 1.21)**	1.24(1.15, 1.34)**
**Model 3**	1.09(1.02, 1.17)*	1.05(0.97, 1.14)	1.20(1.07, 1.34)**
**VIM (+6.021 mmHg)**			
**Model 1**	1.14(1.05, 1.23)*	1.10(1.01, 1.20)*	1.32(1.11, 1.58)*
**Model 2**	1.15(1.06, 1.26)*	1.11(1.01, 1.22)*	1.38(1.14, 1.66)*
**Model 3**	1.18(1.08, 1.28)**	1.13(1.03, 1.25)*	1.40(1.16, 1.68)**

+: represents the per SD increment of variability

Model 1: adjusted for age, gender, and body mass index;

Model 2: further adjusted for current smoking, current drinking, education level, income level, physical activity, heart rate, diabetes mellitus, dyslipidemia, serum uric acid, high-sensitivity C-reactive protein after logarithmic transformation, history of MI, family history of stroke, and the use of antihypertensive drugs;

Model 3: further adjusted for mean of systolic blood pressure and mean of diastolic blood pressure.

*$P < 0.05$, **$P <0.001$.

TABLE S2. Hazard ratios for stroke by quartiles of coefficient of variation of diastolic blood pressure and per SD increases in the coefficient of variation#

	**Quartiles of CV of DBP**				**CV of DBP**
	**Q1 (<5.21%)**	**Q2 (5.21% to <7.30%)**	**Q3 (7.30% to <11.06%)**	**Q4 (≥11.06%)**	**(per SD increase)**
**Any stroke**					
**Case (%)**	128(0.94)	130(0.96)	139(1.04)	185(1.36)	
**Model 1**	1.00 (ref)	0.98(0.77, 1.27)	1.13(0.88, 1.45)	1.39(1.10, 1.75)*	1.16(1.07, 1.26)**
**Model 2**	1.00 (ref)	0.96(0.73, 1.26)	1.16(0.89, 1.51)	1.30(1.01, 1.68)*	1.14(1.05, 1.24)*
**Model 3**	1.00 (ref)	0.95(0.72, 1.25)	1.17(0.90, 1.53)	1.29(1.00, 1.66)*	1.11(1.02, 1.21)*
**Ischemic stroke**					
**Case (%)**	116(0.85)	107(0.79)	117(0.87)	149(1.10)	
**Model 1**	1.00 (ref)	0.88(0.67, 1.15)	1.06(0.81, 1.37)	1.23(0.96, 1.57)	1.12(1.03, 1.22)*
**Model 2**	1.00 (ref)	0.83(0.62, 1.12)	1.08(0.81, 1.43)	1.15(0.87,1.50)	1.10(1.00, 1.21)*
**Model 3**	1.00 (ref)	0.81(0.60, 1.09)	1.09(0.82, 1.44)	1.10(0.84, 1.44)	1.07(0.98, 1.18)
**Hemorrhagic stroke**					
**Case (%)**	12(0.09)	24(0.18)	22(0.16)	36(0.27)	
**Model 1**	1.00 (ref)	2.28(1.09, 4.77)*	1.94(0.90, 4.18)	3.15(1.55, 6.39)*	1.40(1.16, 1.68)**
**Model 2**	1.00 (ref)	2.76(1.18, 6.51)*	2.26(0.93, 5.49)	3.36(1 .46, 7.72)*	1.37(1.12, 1.68)*
**Model 3**	1.00 (ref)	2.72(1.16, 6.40)*	2.28(0.94, 5.56)	3.15(1.37, 7.24)*	1.30(1.07, 1.59)*

Case (%): number of events (cumulative incidence %)

Model 1: adjusted for age, gender, and body mass index;

Model 2: further adjusted for current smoking, current drinking, education level, income level, physical activity, heart rate, diabetes mellitus, dyslipidemia, serum uric acid, high-sensitivity C-reactive protein after logarithmic transformation, history of MI, family history of stroke, and the use of antihypertensive drugs;

Model 3: further adjusted for mean of systolic blood pressure and mean of diastolic blood pressure.

Per SD increase in the CV of DBP= 4.7%.

*$P < 0.05$, **$P < 0.001$.

TABLE S3. Hazard ratios for stroke with per SD increases in other indicators of variability of diastolic blood pressure

	**Any stroke**	**Ischemic stroke**	**Hemorrhagic stroke**
**SD (+4.111 mmHg)**			
**Model 1**	1.26(1.18, 1.36)**	1.21(1.12, 1.31)**	1.54(1.31, 1.81)**
**Model 2**	1.22(1.12, 1.32)**	1.16(1.07, 1.27)*	1.48(1.24, 1.77)**
**Model 3**	1.10(1.02, 1.19)*	1.07(0.98, 1.17)	1.28(1.07, 1.54)*
**ARV (+5.665 mmHg)**			
**Model 1**	1.17(1.08, 1.26)**	1.13(1.04, 1.22)*	1.36(1.16, 1.61)**
**Model 2**	1.12(1.04, 1.21)*	1.08(0.99, 1.18)	1.33(1.12, 1.58)*
**Model 3**	1.03(0.95, 1.12)	1.00(0.91, 1.09)	1.18(0.98, 1.41)
**VIM (+3.962 mmHg)**			
**Model 1**	1.11(1.03, 1.21)*	1.08(0.98, 1.18)	1.32(1.09, 1.60)*
**Model 2**	1.10(1.01, 1.20)*	1.07(0.97, 1.17)	1.30(1.06, 1.61)*
**Model 3**	1.11(1.02, 1.21)*	1.07(0.98, 1.18)	1.31(1.07, 1.62)*

+: represents the per SD increment of variability

Model 1: adjusted for age, gender, and body mass index;

Model 2: further adjusted for current smoking, current drinking, education level, income level, physical activity, heart rate, diabetes mellitus, dyslipidemia, serum uric acid, high-sensitivity C-reactive protein after logarithmic transformation, history of MI, family history of stroke, and the use of antihypertensive drugs;

Model 3: further adjusted for mean of systolic blood pressure and mean of diastolic blood pressure.

*$P < 0.05$, **$P < 0.001$.
